# Rapid Ex-Vivo Ciliogenesis and Dose-Dependent Effect of Notch Inhibition on Ciliogenesis of Respiratory Epithelia

**DOI:** 10.3390/biom10081182

**Published:** 2020-08-14

**Authors:** Maliha Zahid, Timothy N. Feinstein, Anthony Oro, Molly Schwartz, Alex D. Lee, Cecilia W. Lo

**Affiliations:** Department of Developmental Biology, University of Pittsburgh School of Medicine, 530 45th St, Pittsburgh, PA 15201, USA; maz7@pitt.edu (M.Z.); tnf8@pitt.edu (T.N.F.); anthonyoroperez@gmail.com (A.O.); mollycs23@hotmail.com (M.S.); alexlee921107@gmail.com (A.D.L.)

**Keywords:** cilia, ciliogenesis, siRNA knockdown, Notch signaling, N-[N-(3,5-Difluorophenacetyl)-L-alanyl]-S-phenylglycine t-butyl Ester (DAPT)

## Abstract

**Background**: Cilia are actin based cellular protrusions conserved from algae to complex multicellular organisms like Homo sapiens. Respiratory motile cilia line epithelial cells of the tracheobronchial tree, beat in a synchronous, metachronal wave, moving inhaled pollutants and pathogens cephalad. Their role in both congenital disorders like primary ciliary dyskinesia (PCD) to acquired disorders like chronic obstructive pulmonary disease (COPD) continues to evolve. In this current body of work we outline a protocol optimized to reciliate human nasal epithelial cells and mouse tracheal cells in vitro. Using this protocol, we knocked down known cilia genes, as well as use a small molecule inhibitor of Notch, N-[N-(3,5-Difluorophenacetyl)-L-alanyl]-S-phenylglycine t-butyl Ester (DAPT), to assess the effect of these on ciliogenesis in order to show the validity of our protocol. **Methods**: Tracheas were harvested from wild-type, adult C57B6 mice, pronase digested and sloughed off epithelial cells grown to confluence in stationary culture on rat-tail collagen coated wells. Upon reaching confluence, collagen was digested and cells placed suspension culture protocol to reciliate the cells. Using this suspension culture protocol, we employed siRNA gene knockdown to assay gene functions required for airway ciliogenesis. Knock down of Dynein axonemal heavy chain 5 (Dnah5), a ciliary structural protein, was confirmed using immunostaining. Mouse tracheal cells were treated in suspension with varying doses of DAPT, an inhibitor of Notch, with the purpose of evaluating its effect and dose response on ciliogenesis. The optimum dose was then used on reciliating human nasal epithelial cells. **Results**: siRNA knockdown of *Foxj1* prevented ciliation, consistent with its role as a master regulator of motile cilia. Knockdown of *Dnai1* and *Dnah5* resulted in immotile cilia, and *Cand1* knockdown, a centrosome protein known to regulate centrosome amplification, inhibited airway ciliogenesis. Dnah5 knockdown was confirmed with significantly decreased immunostaining of cilia for this protein. Inhibiting Notch signaling by inhibiting gamma secretase with DAPT enhanced the percentage of ciliation, and resulted in longer cilia that beat with higher frequency in both mouse and human airway epithelia. **Conclusions**: Modifying existing reciliation protocols to suit both human nasal epithelial and mouse tracheal tissue, we have shown that knockdown of known cilia-related genes have the expected effects. Additionally, we have demonstrated the optimal dosage for significantly improving reciliation of airway epithelia using DAPT. Given that cilia length and function are significantly compromised in COPD, these findings open up interesting avenues for further exploration.

## 1. Introduction

Respiratory health depends on muticiliated cells that line the respiratory tract and beat in a metachronal fashion to keep airways clear of infectious microbes, cell debris, and toxic environmental contaminants. This key normal ciliary function is lost in congenital diseases like primary ciliary dyskinesia (PCD), or in acquired conditions like chronic obstructive pulmonary disease (COPD) [1,2] as a result of damage from chronic tobacco smoke inhalation. COPD is a deadly and debilitating respiratory illness with few effective treatment options [3,4]. Indeed, COPD is now the third leading cause of mortality worldwide [5], and this is projected to increase in the coming decades [6,7]. Despite the urgent need for therapies to treat COPD and other ciliopathies, no drug therapy targeting the pathophysiology has been approved for COPD, with gene therapy treatments remaining purely theoretical. The current standard of care is based on use of inhaled corticosteroids to blunt inflammation and bronchodilators to address the obstruction to airflow that is the hallmark of this disease. In view of the important role that cilia play in a wide spectrum of human diseases, development of platforms for efficient high throughput screens in order to identify therapeutic targets and drugs to treat ciliopathies is of considerable clinical importance.

Given the extensive similarities in the mechanism of primary and motile cilia formation, it is not surprising that mutations that affect primary cilia could also perturb the formation or function of motile cilia. Supporting this, several clinical studies have found respiratory airway clearance defects in patients with congenital primary ciliopathies such as Sensenbrenner syndrome [8] or Leber congenital amaurosis [9]. Previous work from our laboratory and others have found a high prevalence of respiratory ciliary dysfunction in patients with complex congenital heart disease [10,11,12]. This was observed in patients with and without heterotaxy [11], and was correlated with a high prevalence of chronic respiratory symptoms and disease [10], with increased post-operative pulmonary morbidity [13,14].

Here we report the use of an in vitro ciliogenesis protocol using isolated cells from mouse trachea as well as epithelial cells obtained from human nasal scrapes. Our protocol is adapted from a method previously described for culturing human nasal epithelial cells [15,16,17]. We used this protocol for the functional assessment of gene knockdown analyses. We also tested the potential of a gamma secretase/Notch inhibitor, *N*-[*N*-(3,5-Difluorophenacetyl)-L-alanyl]-S-phenylglycine t-butyl ester (DAPT) to enhance ciliation in a dose-dependent fashion, and the downstream gene expression consequences of this inhibition.

## 2. Methods

### 2.1. Isolation of Mouse Trachea and Human Nasal Epithelia

All animal protocols were approved by the University of Pittsburgh’s Institutional Animal Care and Use Committee (IACUC-protocol ID number 18124046). All methods were performed in accordance with relevant guidelines and regulations set forth by the IACUC. Adult 6–8 week old C57B6 mice were euthanized, neck region sprayed with 70% ethanol and a transverse incision made over the mid-neck ventral surface. The subcutaneous fat was removed and tracheas exposed, harvested, cleaned of adhering muscle and fat tissue, and splayed length-wise (Supplementary Figure S1) for further processing (see below). All human samples were obtained after informed consent under a University of Pittsburgh Institutional Review Board approved protocol. Nasal ciliated epithelial cells were obtained by scraping the inferior nasal turbinate with a rhino probe under direct visualization with the aid of a nasal speculum. Rhino probe (Arlington Scientific, Springville, UT, USA, Catalog #SY-96-0905), a small, plastic curette was drawn gently over the surface of the inferior nasal turbinate several times. The cells obtained were placed into 5 mL of Leibovitz 15 medium, high-speed video-microscopy performed, and cells immediately placed into culture.

#### Stationary Phase Culturing

**Figure 1:**
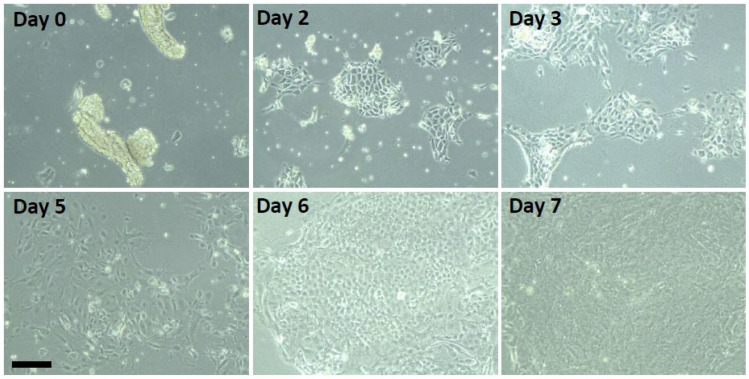
Course of mouse tracheal epithelial cells in culture. Mouse tracheal cells (MTCs) from a single trachea per well were placed on rat-tail collagen coated six-well culture dishes (scale bar represents 50 μm). On Day 0, the cells can be seen as sheets with beating, motile cilia. The cells take 24–48 h to become adherent, loose their cilia, and start dividing. Initial islands of cells form by day 2, expand over the ensuing 4–5 days, becoming confluent by Day 7.

Mouse tracheas were digested with Pronase (1.5 mg/mL; Protease from *Streptomyces griseus*, Sigma, St. Louis, MO, USA, P5147) overnight, at 4 °C leading to release of mouse epithelial tracheal cells (MTCs), followed by neutralization with 10% fetal bovine serum, vortexed for 5–10 s vigorously and spun down. The resulting pellet and tracheas were washed twice with stationary media (DMEM/F12 (Sigma, St. Louis, MO, USA, D6421), 2% Ultroser G (PALL Life Sciences, New York, NY, USA, 15950-017), 10 mL of Antibiotics-Antimycotic (Gibco, Gaithersburg, MD, USA, 15240)). The MTCs along with the digested tracheas were pre-plated for 4 h to allow attachment and removal of contaminating fibroblasts. Non-adherent cells were collected, pelleted, resuspended in stationary medium and plated onto 6-well plates coated with rat tail collagen in a final volume of 2 mL per well. After ~48 h of incubation, cells became adherent, formed a monolayer and exhibited proliferative growth (Supplementary Figure S2). Cells were fed with stationary media every 2 days. In the results of the experiments presented below, one trachea per well of a 6-well plate was used and reached confluence by 5–7 days (Figure 1). As little as one-half trachea per well has been used with resulting increase in time to confluence to 7–10 days.

### 2.2. Suspension Culture for Ciliogenesis

Confluent cultures had stationary medium aspirated and replaced with 1 mL of Collagenase IV (200 IU/mL, Worthington, Lakewood, NJ, USA, LS004210) and returned to incubator for 75 min until all of the collagen layer had dissolved. At the end of the incubation period, collagenase was neutralized with 2 mL of suspension media (500 mL DMEM/F12 (Sigma, St. Louis, MO, USA, D6421), 50 mL NuSerum (Corning, Corning, NY, USA, CB-5500), 10 mL Antibiotics-Antimycotic (Gibco, Grand Island, NY, USA, 15240)). The cells were gently scraped off with a cell scraper (Fisherbrand, Boston, MA, USA, 08-100-241), and came off as large sheets of cells, were pelleted, washed twice with suspension media, resuspended in 12 mL of suspension media and placed in a T-25 flask with non-rebreathing caps. Cells from one six-well plate were added to a T-25 flask, and placed on a heated orbital shaker set at 37 °C with 80-rpm rotation, without any replacement of media over the ensuing 8 days in suspension culture. The T-25 flasks were placed at an angle with the neck end raised at a ~20° angle to prevent sludging of the spheroids in the neck or cap of the flask. Over time, the sheets of cells formed “spheroids” consisting of multiple cells, the exposed surface of which ciliated by day 5–6 with initially nonmotile, short cilia, achieving motility by day 7–8, with ciliogenesis complete by day 8–9. These spheroids remained stable in suspension under constant agitation up to day 9. Beyond 9 days in suspension culture, cells were seen to lose their cilia and become apoptotic. After imaging, the reciliated spheroids from Dnah5 knockdown were fixed in 4% paraformaldehyde (PFA) in phosphate buffered saline (PBS), rinsed, and blocked for 1hr in blocking buffer (1% bovine serum albumin and 0.2% Triton X-100 in PBS), and immunostained overnight using anti-acetylated tubulin, anti-gamma tubulin, anti-Dnah5, and DAPI. Stained organoids were sandwiched under a coverslip on a glass-bottom dish, imaged on a Leica SP8 confocal microscope, and then deconvolved using Huygens software (SVI Inc., DeKalb, IL, USA). We chose Dnah5 for immunostaining due to this gene being well established as a PCD gene and due to the availability of reliable monoclonal antibodies for it.

To assess the effect of Notch inhibition with DAPT (Selleckchem, Houston, TX, USA, S2215) on ciliogenesis, varying concentration of DAPT (2 nM–2 μM) was added to the flask at the suspension stage, with one confluent 6-well plate added to a T-25 flask for each dose of DAPT tested. These dose response DAPT experiments were carried out in biological triplicates. Level of ciliogenesis, ciliary beat frequency and cilia length were calculated from a minimum of 6 videos for each sample by a reviewer (AO) blinded to the treatment status.

Mouse tracheal cells were collected before going into suspension culture (Baseline) and also at days 1, 3, 5 and 8 for RNA extraction and real time polymerase chain reaction (PCR) for analysis of *eNos*, *iNos* and *nNos* transcript abundance (Supplementary Table S1), with all samples normalized to β-Actin. As human nasal epithelial cells were limited in quantity (one sample scrape being placed into 3-wells of a 6-well plate), they were placed only into two T-25 flasks with and without 2 nM DAPT. This dose was selected as the optimum dose for ciliogenesis based on the above MTC experiments.

### 2.3. Assessing Ciliogenesis and Ciliary Motion

**Figure 2:**
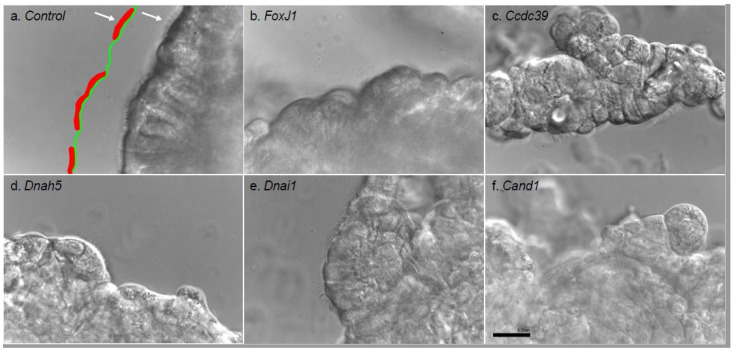
Quantification of ciliation using ImageJ program. Video clips are opened as a single still frame in ImageJ and the circumference of the surface of spheroids that could ciliate is outlined (**a**—red, as well as the actual circumference that achieved ciliogenesis (**a**—green). Dividing the reciliated surface by the total surface provides percent ciliation. Panels b-f show ciliation in suspension after siRNA knockdown of *Foxj1* (**b**), *Ccdc39* (**c**), *Dnah5* (**d**), *Dnai1* (**e**), and *Cand1* (**f**). Scale bar represents 100 μm.

Motile cilia function was assessed using high-speed (200 fps) and high magnification (60×) video-microscopy (Figure 2a). A minimum of six videos were analyzed per sample. The degree of ciliation was calculated using ImageJ (https://imagej.nih.gov/ij/, National Institute of Health, Bethesda, MD, USA) [18] (Figure 2b). The percent ciliation was analyzed in ImageJ by measuring the length of the surface that could possibly reciliate (Figure 2a—green line) and used as the denominator with the surface that ciliated measured (Figure 2a—red line) and used as the numerator in the calculation. To measure cilia length, a custom script was written to segment patches of cilia based on movement, with the thickness tool in the BoneJ analysis toolkit [19] adapted to measure average cilia length (see Supplementary Methods for detailed script). 

The day before gene knockdown, stationary media was replaced with 2.0 mL of antibiotic-free stationary medium, which was aspirated and replaced with 1.7 mL of antibiotic-free stationary media on the day of the knockdown. A cocktail of 4 siRNAs (Qiagen FlexiTube siRNA) for the gene of interest (2.5 μL of a 10 μm stock solution each) was added to 140 μL of Opti-MEM (Life Technologies, Rockville, MD, USA, 31985-062). A second mix of 7.5 μL of Lipofectamine RNAiMax (Invitrogen, 13778030) was added to 142.5 μL of Opti-MEM. The two siRNA and Lipofectamine cocktails were incubated together for 5 min at room temperature and added to each well resulting in a final volume of 2 mL. Scrambled siRNA was used as control. The cells were incubated with siRNA for 3 h, followed by collagenase digestion and protocol for placing cells in suspension as above followed. A sample of cells at the time of placement into suspension culture was obtained to assess for knockdown efficiencies. RNA from cells was extracted (RNeasy Plus Micro Kit; Qiagen, Hilden, Germany, 74134), converted to cDNA (High-Capacity RNA to cDNA Kit; ThermoFisher, Waltham, MA, USA, 4387406), and real time PCR performed (primers used listed in Supplementary Table S2). All data was normalized to β-actin. The degree of ciliation was assessed as detailed above. Ciliary motion was assessed by three reviewers, with extensive experience in cilia motion assessment, blinded to the experimental conditions.

### 2.4. Statistical Analyses

Data was tested for normality using the skewness and kurtosis normality test. Normally distributed data were compared using the Student’s unpaired *t*-test. Non-normally distributed data were compared using the Wilcoxon-rank sum test. Two-tailed *p*-value of <0.05 was considered significant with all analyses performed using Stata 12.1 (College Station, Texas, TX, USA).

### 2.5. Results

#### 2.5.1. A Suspension Culture Method for Measuring Mouse Airway Cell Reciliation

Air–liquid interface (ALI) cultures are a common method for reciliation studies, but media requirements are laborious and expensive posing significant challenges to scaling up for screening studies. We therefore adapted a suspension culture method to facilitate studies of reciliation at a higher throughput that is more amenable to genetic screening and dose response curves. Tracheas were isolated from adult mice (Supplementary Video S1), digested in pronase to release MTCs as clusters that continued to show ciliary beating (Supplementary Video S2). These were placed in culture for adherent growth and within 48 h, large islands of cells could be observed attached to the culture dish substratum (referred to as stationary culture). These epithelial cells dedifferentiated, lost their cilia, entered a rapid proliferative phase, and reached confluence by ~5–7 days (Figure 1). In suspension culture, constant agitation triggered robust ciliogenesis in 8–9 days (Supplementary Video S3). It should be noted that at this stage, cells were not fed over the course of 8 days. Additionally, use of a non-breathing flask top was essential as using a filter top resulted in no ciliation (data not shown). The total time from tracheal harvest to completion of ciliogenesis was achieved in ~2 weeks. Our protocol led to successful reciliation of MTCs >90% of the time.

To reduce fibroblast contamination in the tracheal isolates, a pre-plating step was carried out after pronase treatment to remove the more rapidly attaching fibroblast cells. Quantitation of transcripts for fibroblast-specific protein (*Fsp1*), a fibroblast marker [20], showed efficacy of the pre-plating steps in removing most of the contaminating fibroblasts from the explanted cultures (Supplementary Figure S3). Pre-plating onto Primaria culture plates with proprietary extracellular matrix coating did not reduce fibroblast contamination more effectively than regular culture plates (Supplementary Figure S3).

This modified ex vivo ciliogenesis protocol can also be used to achieve rapid ciliation of human nasal epithelial cells obtained from nasal scrapes. Differing from the mouse tracheal explants, the pronase digestion step is not needed, since the nasal epithelia obtained from nasal scrapes is comprised mostly of a single layer of ciliated epithelia. As the number of ciliated cells obtained from human nasal scrapes is significantly lower than from the mouse tracheal explants, growth to confluence usually takes much longer (~2–3 weeks). Additionally, as the number of cells is limited, they are placed in only 3 wells of a 6-well plate. However, once placed into suspension culture, time to ciliation is 7–9 days, similar to the MTCs.

#### 2.5.2. Evaluation of Genetic Contribution to Reciliation through siRNA Knockdown

**Figure 3:**
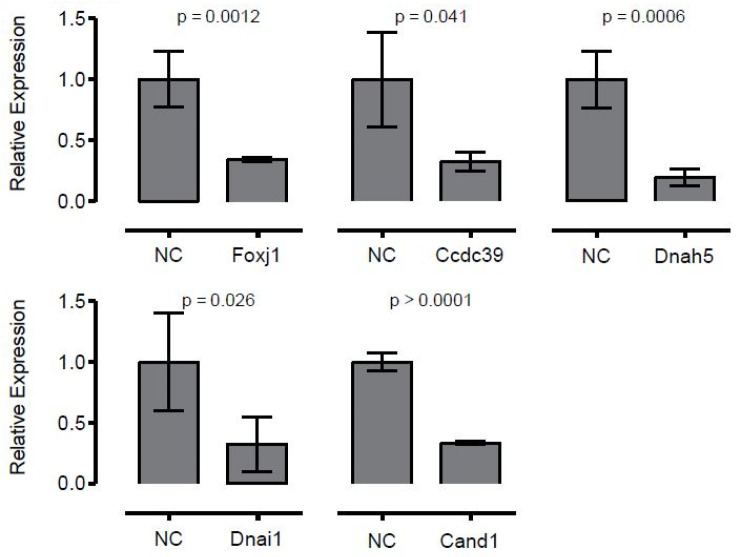
Knockdown of *Foxj1*, *Ccdc39*, *Dnah5*, *Dnai1* and *Cand1* by siRNA shows significant reduction in expression of these genes as compared to negative control, scrambled siRNA (NC). This knockdown is apparent immediately (6 h) post-knockdown. All expression levels were normalized to actin. Error bars represent ± 1 SD.

Gene knockdown with siRNA treatment of the ciliating tracheal epithelial cultures was conducted to assess genes essential for airway ciliogenesis. As a positive control we used siRNA knockdown of *Foxj1*, a transcription factor thought to be the master regulator of ciliogenesis in motile cilia [21], alongside scrambled siRNA as a negative control. Real time PCR analysis of the *Foxj1* vs. scrambled siRNA treated epithelia showed significant reduction of *Foxj1* transcripts by 6 h after siRNA treatment in the *Foxj1* siRNA treated explant (Figure 3). While control cultures had robust ciliogenesis with normal ciliary motion (Supplementary Video S4), *FoxJ1*-depleted explants showed marked inhibition of ciliogenesis and the few patches of cilia observed were largely immotile (Table 1) (Supplementary Video S5). Very few motile cilia were observed; among those the length and beat frequency were similar to control, indicating these were likely cells that escaped siRNA knockdown. As further proof of principal, we knocked down two PCD-associated genes that encode proteins in the outer dynein arm of motile cilia, *Dnai1* and *Dnah5*, and again validated the knockdown with RT-PCR (Figure 3). There was no significant change in ciliogenesis or cilia length with *Dnah5* or *Dnai1* knockdown (Table 1); however, as expected approximately half of ciliated cells were immotile. Where motile cilia were present, the ciliary beat frequency was normal, though some motile cilia exhibited dyskinetic ciliary motion (Supplementary Video S6 and S7). Finally, we knocked down a less well-characterized protein required for assembly of dynein regulatory and inner dynein arm complexes, *Ccdc39* [22] (Figure 3). *Ccdc39* siRNA treatment significantly inhibited ciliogenesis. The few cilia generated were very short and none exhibited motility.

**Figure 4:**
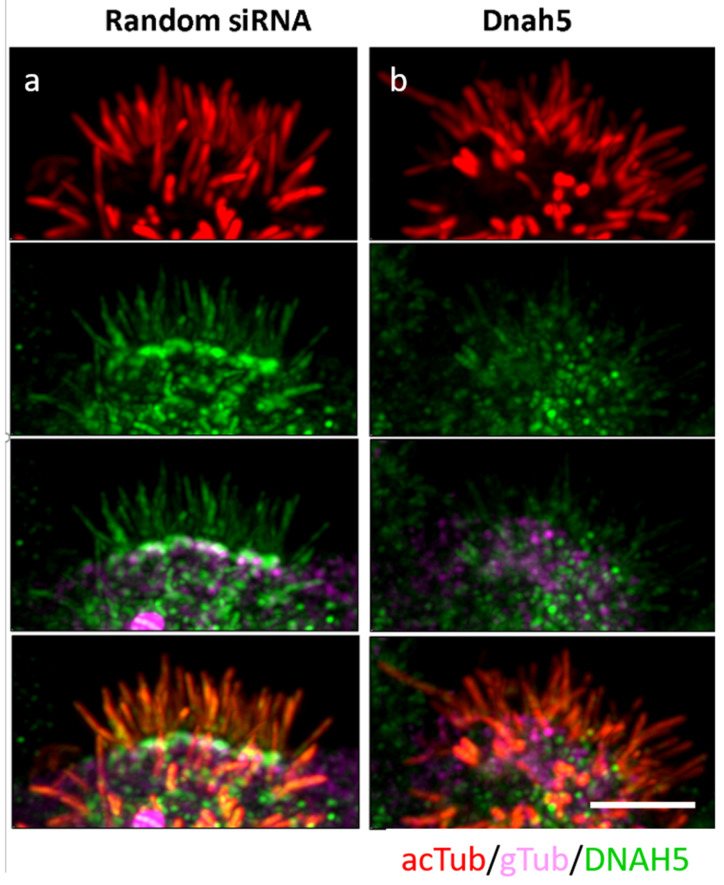
Immunofluorescence staining of reciliated spheroids treated with scrambled, random siRNA (**a**) or Dnah5 knockdown (**b**). Cells were fixed and stained for acetylated tubulin (cilia-top row-red), gamma tubulin (basal body-purple), and Dnah5 (green). In the siRNA treated sample, there is expression of Dnah5 (green) in cilia (red). This expression is significantly decreased in poorly ciliated Dnah5 knockdown spheroids (**b**). Scale bars represent 4 microns.

To further explore the use of this ex vivo airway ciliogenesis assay to assess gene function required for ciliogenesis, we examined the effects of siRNA gene knockdown on centrosome protein, *Cand1*. *Cand1* has been shown to control stability of Plk4, a master regulator of centriole biogenesis that plays an essential role in multiciliation of cells with motile cilia [23]. *Cand1* siRNA knockdown largely blocked ciliogenesis, though the few cilia observed had normal ciliary beat frequency, indicating again that these cells had likely escaped siRNA gene knockdown. In order to confirm knockdown, in addition to qPCR, reciliating spheroids treated with scrambled siRNA (Random) and Dnah5 were immunostained for acetylated tubulin (cilia marker), gamma tubulin (basal body), and Dnah5. Dnah5 was seen to co-localize with both cilia and the basal body in the random siRNA treated sample (Figure 4a). This staining was diminished significantly in the cilia seen in the Dnah5 siRNA treated sample (Figure 4b), confirming that our siRNA knockdown protocol had indeed worked.

#### 2.5.3. Enhancing Ciliogenesis with Notch Inhibition

**Figure 5:**
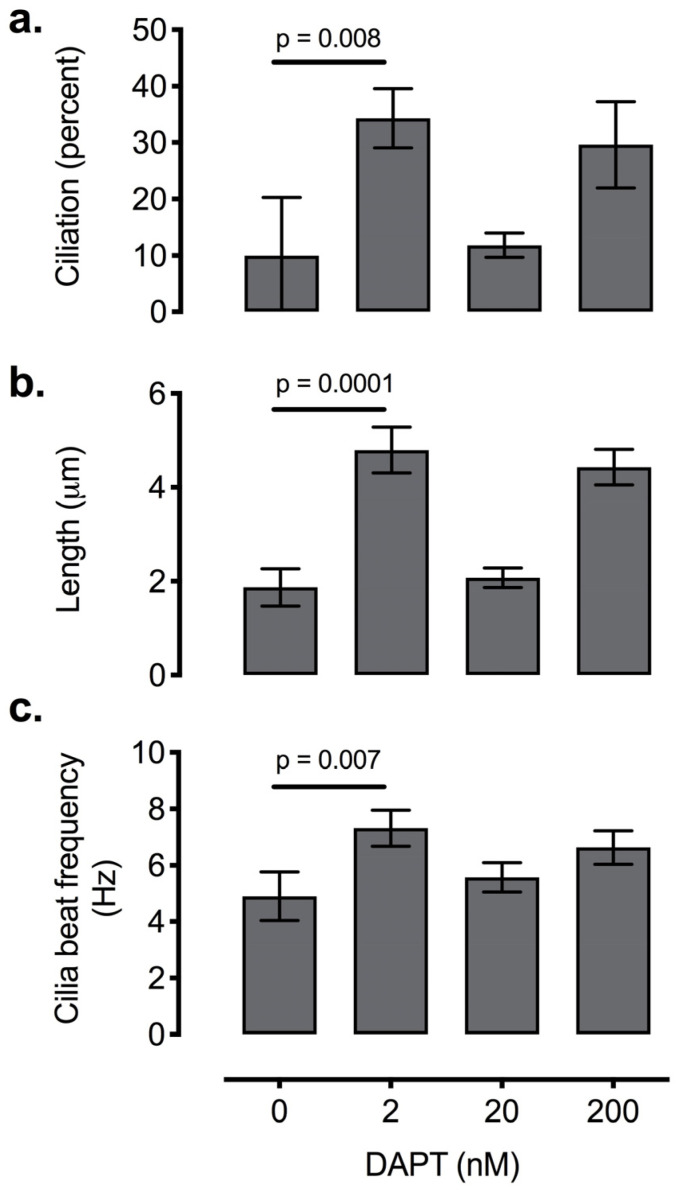
Effect of varying concentrations of DAPT, a Notch antagonist, on ciliation of mouse tracheal epithelial cells (**a**). Addition of 2 nM of DAPT resulted in ~40% greater degree of ciliation over control media. However, concentrations of DAPT at 2 μM were cytotoxic. DAPT 2 nM also significantly increased cilia length (**b**) and ciliary beat frequency (**c**). Error bars represent ± 1 SD.

Finally, we assayed the potential of Notch pathway inhibition for enhancing ciliogenesis as potential therapy to be tested in future, experimental models of CODP. The Notch pathway influences motile ciliogenesis [24,25], and gamma secretase inhibition via DAPT has been used as part of ALI culture reciliation studies [26,27]. We sought to test the therapeutic potential of DAPT by assessing its effective dosage. We examined the effects of treatment with varying doses of DAPT (2 nM–2 μM), a gamma-secretase inhibitor that prevents cleavage of the intracellular domain of Notch driving the downstream transcriptional effects of Notch signaling. The minimum dosage of 2 nM DAPT increased ciliogenesis by almost three-fold over cultures treated with DMSO vehicle alone (Figure 5). However, increasing DAPT concentrations did not saturate the positive response. Instead, ciliogenesis at 20 nM was equivalent to baseline levels. Ciliation then increased again at 200 nM DAPT. Dosages of 2 μm or greater proved lethal to cells in three out of three trials. Furthermore, 2 nM DAPT significantly enhanced cilia length and ciliary beat frequency (Figure 5b,c).

**Figure 6:**
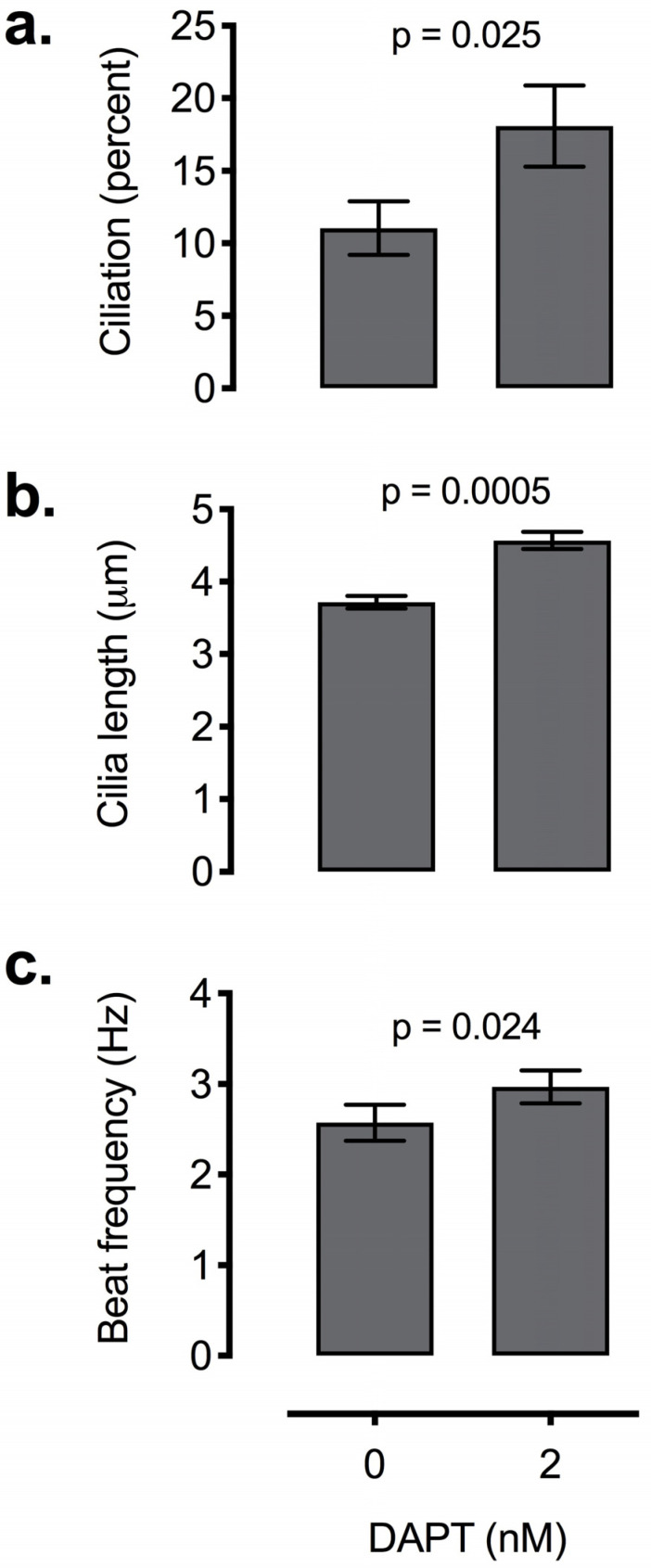
Effect of 2 nM of DAPT on ciliation of human nasal epithelial cells from 12 subjects. DAPT treatment led to significant increase in ciliogenesis (**a**), cilia length (**b**) as well as ciliary beat frequency (**c**). Error bars represent ± 1 SEM.

We next performed the same DAPT treatment at a dose of 2 nM on epithelial cultures isolated from 12 independent human nasal epithelia samples. As in MTC culture, 2 nM DAPT increased ciliogenesis, cilia length and CBF in reciliating human nasal epithelia (Figure 6a: *p* < 0.0001). In addition, both cilia length and ciliary beat frequency were also significantly increased with the 2 nM DAPT treatment (Figure 6b,c: *p* < 0.0001). The variance in magnitude of percent reciliation, cilia length and CBF was strikingly small between subjects, demonstrating that this dosage of DAPT is beneficial across species and diverse genetic backgrounds (Supplementary Figure S4). Due to limited availability of material in human samples, a dose response curve for DAPT with human nasal epithelial scrapes was not possible.

**Figure 7:**
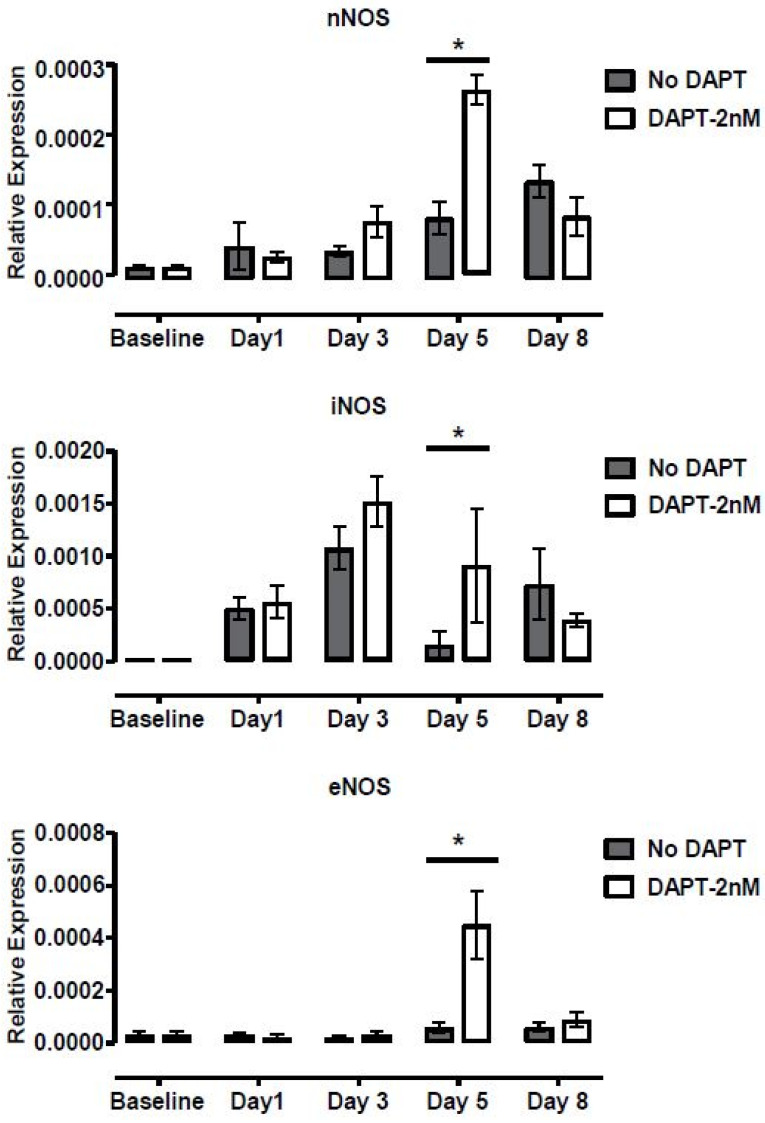
Effect of 2 nM of DAPT on Nos expression. Treatment of MTCs in suspension with 2 nM of DAPT led to significant increase in *iNos*, *eNos*, and *nNos* gene expression as compared to586 untreated, control samples on Day 5. All data was normalized to mouse β-actin. * represents two-tailed *p* < 0.001.

To test whether DAPT enhances motile ciliogenesis through inhibition of Notch signaling, real time PCR for *Notch1* and downstream Notch regulated genes was carried out. These studies showed *Notch1* transcript level was not affected by DAPT treatment during the induction of ciliogenesis in suspension culture, but surprisingly, by day 5 of ciliogenesis the expression of Notch effector genes *Hes5* and *Hey1* were significantly elevated (Supplementary Figure S5). Notably, the expression of all three isoforms of *Nos* increased significantly on Day 5 in DAPT treated samples compared to controls treated with DMSO alone (Figure 7).

## 3. Discussion

At the same time that mortality from cardiovascular diseases and cancer has steadily decreased over time, mortality from chronic respiratory disorders has not changed in the last two decades and is projected to be the leading cause of mortality by year 2030 [28]. COPD remains a major threat to human health with few treatment options available [1,2,29]. Yet, no new drugs have reached the market that target the underlying pathophysiological mechanisms of COPD largely due to incomplete understanding of the pathways leading to this common, chronic, debilitating disease. In this study, we used an in vitro reciliation protocol to measure the production of motile cilia in mouse and human airway epithelia, in order to test the role of several genes in reciliation by way of siRNA knockdown as well as small molecule inhibitors. Our protocol, adapted from protocols used since the 1980s [30,31,32,33,34], is highly reproducible and results in robust ciliation that is complete in ~2 weeks for MTCs. Using, on average, one trachea per well of a 6-well plate, with an entire plate going into one suspension culture, confluence was reached in stationary culture in 5–7 days. We could not quantify the number of cells going into suspension culture as the cells came off as large sheets upon collagenase treatment and by day 8 in suspension had formed round organoids, that we termed spheroids. Overall, as is often the case with mouse experimental models, it is far more practical to use MTCs, with many mouse models of PCD and other respiratory diseases readily available [35], making them practical for drug discovery screening. Human samples were also viable in this model, but needed more time to reach confluence due to limited number of cells available from each individual nasal scrape.

Relative to ALI protocols, the model described here has much simpler media requirements, with only Ultroser-G or NuSerum needed for stationary and suspension culture medium, respectively, as opposed to multiple antibiotics, antifungals, growth factors and vitamins that add to the complexity and reduce the screening potential of ALI methods [33,36,37]. Ultroser-G is imported from France and requires a license from the United States Department of Agriculture (USDA), which can be readily obtained online at: https://www.aphis.usda.gov/aphis/ourfocus/animalwelfare/SA_Regulated_Businesses/SA_Request_License_Registration_Application_Kit. We note that one study based on ALI cultures reported a success rate of 65% [38] while our protocol resulted in ciliogenesis over 90% of the time. However, we did not conduct a head-to-head comparison between the two methodologies as that was not the focus of the paper and our early results with ALI culture protocol had been poorly reproducible.

We did not assess for mucin producing goblet cells or mucin-related genes (Muc2a, Muc5ac, etc.), as our focus was on ciliogenesis, though it would be simple to either stain mature, Day 8–9 spheroids for mucin or use real time PCR to assess mucin gene expression. This angle could be important for future endeavors, since pathology associated with diseases such as COPD, asthma or cystic fibrosis can involve increased mucus production and goblet cell hyperplasia in addition to poor ciliary function. To demonstrate the potential of our protocol to measure reciliation, we used siRNA knockdown to assay for the effect of siRNA-mediated knockdown of key ciliogenesis genes. We took aliquots of cells from suspension culture at different time-points but saw the most significant decrease in mRNA expression at 3 h post transfection. This is not surprising as siRNA knockdown is transient and not expected to target all the cells. As expected, knockdown of *Foxj1* and *Cand1* largely inhibited airway ciliogenesis, while *Dnah5* and *Dnai1* knockdown resulted in immotile cilia [21]. FoxJ1 is a key upstream transcriptional activator of ciliation, while Cand1 regulates centriole duplication during multi-ciliogenesis [23], and *Dnah5* [39] and *Dnai1* [40] are part of the outer dynein arm complex and not expected to inhibit ciliogenesis. PCD patients with *Dnah5* or *Dnai1* mutations do not show lack of airway ciliogenesis, but rather exhibit immotile or dyskinetic cilia similar to our observations [39,40]. Reciliated spheroids treated with either scrambled siRNA or siRNA targeting Dnah5 were fixed and stained for acetylated tubulin (cilia marker) and Dnah5. Successful knockdown was confirmed with significantly decreased Dnah5 staining in cilia as compared with scrambled siRNA treated spheroids. Some cytoplasmic, non-specific Dnah5 staining was visible but is consistent with publications in the literature on transgenic Dnah5 mutant mice [41]. Our *Ccdc39* knockdown analysis also showed reduced airway ciliogenesis and cilia immotility, similar to PCD patients harboring *CCDC39/CCDC40* mutations [42,43,44]. We recommend that for testing novel, putative cilia genes, our methods should be used for screening purposes with interesting leads confirmed by Lentiviral transfections, which are more likely to give more complete and sustained knockdown.

Finally, we sought to clarify the role of Notch in the development of airway cilia by measuring reciliation after treatment with DAPT, a small molecule Notch inhibitor. A key regulator of stem cell fate decisions, Notch encourages the development of mucus-secreting Club cells [45] and its inhibition would be expected to encourage differentiation to ciliated epithelia instead. Indeed, Notch inhibition with 2 nM DAPT increased airway ciliogenesis, as well as cilia length and ciliary beat frequency. These ciliogenesis promoting effects of DAPT were observed in both MTCs as well as human nasal epithelial cells. Moreover, all 12 unrelated human subjects responded to 2 nM DAPT, attesting to the robustness of these effects. However, other doses of DAPT did not match a simple sigmoidal dose-response relationship; instead showing striking evidence of bimodality, with baseline levels of ciliogenesis at 20 nM, enhanced ciliogenesis and CBF at 200 nM similar to 2 nM treatments, and extensive cell death at 2 μM. This bimodal response to DAPT was both unexpected and intriguing. A possible explanation is the observation that presenilin, the catalytic component of gamma secretase, localizes to two distinct fractions of cells from the mouse brain cortex [46], suggesting that two functionally distinct populations of gamma secretases exist that could have significantly different DAPT responsiveness. Notably, in contrast with prior reports [27,47], we saw cellular toxicity at doses of 2 μM, suggesting that unanticipated aspects of our protocol may sensitize cells to death via Notch inhibition. This also suggests that the biphasic dose response curve may also be unique to our system, and thus any exploration of DAPT-mediated reciliation in vivo should include thorough dose response testing as well. *Notch1* expression levels did not change during suspension culture or after addition of DAPT media. However, transcripts of several genes downstream from *Notch1*, notably *Hes5* and *Hey1*, were upregulated in 2 nM DAPT on day 5, immediately before the appearance of cilia. As others have shown, Hes5 protein expression is acutely downregulated by Notch inhibition with DAPT [48]; this would suggest increased *Hes5/Hey1* transcript levels might reflect a compensatory feedback loop. These findings suggest that Notch effectors could be promising new targets for developing therapeutics to treat airway diseases that involve pathogenic mucociliary dysfunction. Whether these beneficial effects of DAPT translate into increase in mucociliary clearance in vivo remains to be seen and is an area of active on-going study. In time course studies using 2 nM DAPT versus DMSO control, all three isoforms of *Nos* increased on day 5. For *iNos*, the increase occurred earlier on Day 3, but the difference was most prominent on Day 5. Ciliary function correlates with exhaled nitric oxide (NO) levels; in fact the link is so strong that low exhaled NO is recommended as a screening tool for PCD [49], although the mechanism linking NO to cilia motility remains unknown. Do healthy motile cilia produce more NO, or does NO contribute to the Notch-related pathway by which airway stem cells differentiate preferentially into ciliated cells? If Notch inhibition by DAPT leads to upregulation of all *Nos* isoforms on day 5, prior to appearance of cilia, this suggests that the Nos pathway may contribute to ciliogenesis and is not a downstream consequence of motile cilia. In fact NO has a complicated relationship with Notch, which can be either activating [50], or inhibitory [51] depending on the cellular context. This complex feedback relationship may present an alternative explanation for the complex dose-dependent relationship between Notch and multi-ciliated cells. Further studies are needed to validate these findings and better define the relationship between NO, Notch, and ciliogenesis.

## 4. Limitations

Our study has several limitations. We did not perform a side-by-side comparison of ciliation protocol with ALI culture. This would be especially problematic with human nasal epithelial cultures due to limited tissue sample to work with. We were unable to quantify the number of cells placed in suspension as they came off as large sheets that formed spheroids consisting of dozens to hundreds of cells. For the purpose of real-time PCRs however, all data was normalized to β-actin to control for changes in cell number. We also confirmed the results by immunostaining for Dnah5 in the reciliated knockdown sample and comparing to scrambled siRNA as negative control. Although the rat-tail collagen extraction process requires 5–7 days, the resulting stock solution can be stored for up to 6 months at 4 °C. We did not compare this to commercially available stocks of rat collagen. We anticipate that rat collagen type IV would work equally well. However, we work with a very large number of human nasal epithelial and MTC cultures, which would make commercially available rat collagen prohibitively expensive. Lastly, the ciliary beat frequencies reported for both MTCs and human reciliating cultures are lower than what has been reported in the literature. We imaged these cells at room temperature and not at 37 °C thereby most likely lowering the beat frequencies, as cilia are extremely temperature sensitive [52,53]. Lastly, whether this beneficial effect of DAPT on cilia parameters translates into improved mucociliary clearance in vivo remains to be seen, and is an area of active study in our lab.

## 5. Conclusions

Our adapted protocol was used to demonstrate the feasibility of studying the complex process of ciliogenesis in both mouse tracheal and human nasal epithelial cells. We validated this by knocking down known genes key for ciliogenesis and observing the expected outcome. This has the potential for use in genetic and small molecule screens for identifying genes or pathways that could be manipulated to enhance ciliogenesis, and by extension, mucociliary clearance, a process impaired in many key, common pulmonary pathologies. We demonstrate an interesting bimodal effect of Notch inhibitor, DAPT, on ciliogenesis. We note respiratory epithelia are especially promising as a therapeutic target due to the potential for inhalant delivery of aerosolized drugs, an approach that avoids potentially harmful side effects from exposure of non-target organs or systems. This strategy has already been validated for delivering gene therapy vectors to treat cystic fibrosis [54].

## Figures and Tables

**Table 1:** Gene knockdown on respiratory epithelia ciliogenesis.

Gene Knockdown	Ciliation (%)	Cilia Beat Frequency (Beats/sec)	Cilia Length (μm)	Cilia Motion
*Control*	16.3 ± 8.7	7.4 ± 1.1	2.6 ± 0.7	Normal
*Foxj1*	2.4 ± 3.4 *	7.0 ± 0.6	3.3 ± 0.2	Mostly immotile
*Ccdc39*	3.8 ± 7.6 *	0	1.5	Immotile
*Dnah5*	15.0 ± 8.2	7.1 ± 1.1	3.2 ± 1.1	50% immotile
*Dnai1*	10.3 ± 4.0	7.2 ± 1.0	2.1 ± 0.7	50% immotile
*Cand1*	4.0 ± 5.6 *	7.6 ± 1.4	1.7 ± 0.3	Normal

* Two-tailed *p* < 0.05 for comparison to negative control using Wilcoxon rank-sum test.

## Supplementary Materials

The following are available online at https://zenodo.org/record/3988053#.Xzpj4Oe-uUl, Supplementary Methods detailing rat tail collagen extraction, coating of culture plates with collagen, nasal scrapes to obtain human nasal epithelial cells, and analysis of videos of reciliated spheroids. Figure S1: Illustration of step by step protocol for harvesting tracheas from mice, Figure S2: Protocol for knock-down of ciliogenesis using siRNA, Figure S3: Quantification of fibroblast contamination as assessed by transcript levels of fibroblast specific marker Fsp., Figure S4: Reciliation (a), cilia length (b) and ciliary beat frequency (c) from 12 subjects treated with suspension media with and without DAPT, Figure S5: Transcript expression of Notch 1 and genes downstream of Notch1 (Hes1, Hes5, Hey1) at various time points in the ciliation protocol in regular vs. DAPT treated media, Table S1: Primer sequence for real time rt-PCR of Notch1 and genes downstream of Notch1 (Hes1, Hes5, Hey1), Table S2: Primer sequence for real time rt-PCR of all the genes (Foxj1, Ccdc39, Dnah5, Dnai1, Cand1) knockdown using siRNA, Video S1: Tracheal surface with normal ciliated epithelium, Video S2: Persistence of normal ciliated tracheal epithelial tissue after overnight pronase digestion at 4 °C, Video S3: Video-microscopy of mouse tracheal epithelial tissue upon reciliation after 8 days in suspension culture, Video S4: Scrambled siRNA negative control showing robust reciliation at 8 days in suspension culture, Video S5: siRNA knockdown of Foxj1 showing successful knockdown of ciliogenesis with only a few flickering cilia seen, Video S6: siRNA knockdown of Dnah5 showing near-normal ciliation levels but with ~50% immotile cilia, Video S7: siRNA knockdown of Dnai1 showing near-normal ciliation levels but with ~50% immotile cilia.
